# Improving Physicians' Adherence to the American Heart Association Guidelines for Airway Management: A Clinical Audit at Almanagil Teaching Hospital

**DOI:** 10.7759/cureus.113815

**Published:** 2026-08-02

**Authors:** Noha Salaheldeen Taha Mahde, Ibrahim Abdelhafiz Ibrahim Ahmed, Amgad Elhadi Mousa Mohamed, Khawla Abdalrouf Mohammad Khair Suliman, Arwa Eltayeb Ahmed Ibrahim, Mahdi Hamdan Salih Elnemair, Dina Abdelnaser Ahmed Hussein, Mohamed Yasin Humaida Ibrahim, Momen Babiker Eltayab Eltayar, Husameldin Ali Babiker, Aya Ismail Ahmed Mohammedzain, Weam Mohamed Elhadi Bashir Eltayeb, Randa Azhari Badawi Abdelmagid, Aya Abdelfattah Kaml Hassan, Khalid Monawar, Mohammedomer Eltayeb, Malaz Elkhalifa, Mustafa Mohamed, Marwa Yousif Suliman Mohamed, Tala Siefuddin Awadelkarim Mohamed, Omar Abdelmajeed Maryoud Elbashir, Abdalmahmoud Asadig Kanan Ahmed, Samar Ahmed Abdelaziz Ibrahim

**Affiliations:** 1 Medicine, University of Bahri, Khartoum, SDN; 2 Surgery, Almanagil Teaching Hospital, Almanagil, SDN; 3 Pediatrics, National Ribat University, Khartoum, SDN; 4 General Medicine, Assiut University Hospital, Assiut, EGY; 5 Emergency Medicine, Dongola Teaching Hospital, Dongola, SDN; 6 Internal Medicine, Assiut University Hospital, Assiut, EGY; 7 Emergency Medicine, Atbara Teaching Hospital, Atbara, SDN; 8 Internal Medicine, Al Neelain University, Khartoum, SDN; 9 Emergency Medicine, Almanagil Teaching Hospital, Almanagil, SDN; 10 Internal Medicine, Almanagil Teaching Hospital, Almanagil, SDN; 11 General Surgery, Almanagil Teaching Hospital, Almanagil, SDN

**Keywords:** advanced cardiovascular life support, airway management, cardiopulmonary resuscitation, clinical audit, quality improvement

## Abstract

Background

Effective airway management is a critical component of cardiopulmonary resuscitation (CPR) and emergency care. Deficiencies in healthcare professionals' knowledge of and adherence to evidence-based airway management principles may compromise the quality of resuscitation and patient outcomes. This study aimed to evaluate the effectiveness of a multifaceted quality improvement intervention in improving healthcare professionals' knowledge of and adherence to Advanced Cardiovascular Life Support (ACLS) airway management principles through a closed-loop clinical audit.

Methods

A prospective closed-loop clinical audit was conducted at Almanagil Teaching Hospital, Sudan. The first audit cycle was performed over two weeks (1 March-14 March 2026) and included 50 healthcare professionals comprising house officers, medical officers, and registrars. Baseline knowledge of airway management was assessed using a structured questionnaire based on the 2025 American Heart Association (AHA) ACLS guidelines. Following the initial assessment, a one-month quality improvement program incorporating structured educational sessions, practical demonstrations, educational materials, visual reminders, and regular feedback was implemented. A second audit cycle involving 50 participants was conducted over two months (15 April-14 June 2026) using the same methodology. Compliance rates between audit cycles were compared using the chi-square test, with statistical significance defined as p<0.05.

Results

Marked improvements were observed across most assessed airway management domains following implementation of the quality improvement intervention. Significant improvements were demonstrated in minimizing interruptions in chest compressions during intubation (66.0% vs. 96.0%, p 0.001), use of waveform capnography for airway confirmation (66.0% vs. 98.0%, p<0.001), knowledge of the maximum recommended interruption time during CPR (46.0% vs. 98.0%, p<0.001), continuous chest compressions following advanced airway placement (8.0% vs. 68.0%, p<0.001), knowledge of airway adjuncts (42.0% vs. 82.0%, p<0.001), understanding of the significance of end-tidal carbon dioxide (ETCO₂) during CPR (48.0% vs. 88.0%, p<0.001), and leadership during resuscitation (78.0% vs. 98.0%, p=0.002). Although improvements were also observed in bag-valve-mask ventilation, recognition of poor airway management practices, and knowledge of hyperventilation, these changes did not reach statistical significance.

Conclusions

A structured, multifaceted educational intervention implemented through a closed-loop clinical audit was associated with significant improvements in healthcare professionals' knowledge of and adherence to evidence-based airway management principles. Regular clinical audit, targeted education, practical reinforcement, and continuous feedback represent feasible and effective quality improvement strategies for strengthening airway management practices, particularly in resource-limited healthcare settings. Continued re-audit and competency-based training are recommended to sustain these improvements and promote safer emergency care.

## Introduction

Effective airway management is a cornerstone of emergency and critical care medicine and is essential for maintaining adequate oxygenation and ventilation in critically ill patients [1]. During cardiopulmonary resuscitation (CPR), timely airway management contributes to preventing hypoxic brain injury while supporting uninterrupted, high-quality chest compressions [2]. Airway interventions range from basic techniques, including the head tilt-chin lift and jaw-thrust maneuvers, to advanced procedures such as supraglottic airway insertion and endotracheal intubation [1]. Current international resuscitation guidelines emphasize that airway interventions should minimize interruptions in chest compressions and that continuous waveform capnography should be used to confirm and monitor advanced airway placement whenever available [2,3].

The American Heart Association (AHA) Advanced Cardiovascular Life Support (ACLS) guidelines provide evidence-based recommendations for airway management during cardiac arrest and other life-threatening emergencies [2]. These recommendations include early airway assessment, effective bag-valve-mask ventilation, avoidance of excessive ventilation, appropriate timing of advanced airway placement, and continuous monitoring of ventilation after airway insertion. Adherence to these recommendations has been associated with improved quality of resuscitation and better patient outcomes, whereas deviations from guideline-based practice may lead to inadequate ventilation, prolonged interruptions in chest compressions, delayed recognition of misplaced endotracheal tubes, and poorer neurological outcomes [4].

Despite the widespread availability of international airway management guidelines, studies continue to demonstrate considerable variability in healthcare professionals' knowledge, technical skills, and adherence to recommended airway management practices [4]. These deficiencies are particularly evident in resource-limited healthcare settings, where opportunities for simulation-based training and continuing professional development may be limited [5]. Furthermore, airway management competence declines over time without regular practice and reinforcement, emphasizing the importance of continuous education and structured competency assessment [5].

Clinical audit is a well-established quality improvement methodology that enables healthcare organizations to compare current practice with evidence-based standards, implement targeted interventions, and evaluate whether measurable improvements have been achieved through a closed-loop process [6]. Previous airway quality improvement initiatives have demonstrated that structured interventions, including staff training, standardized protocols, and audit with feedback, can significantly improve first-pass intubation success and reduce airway-related complications in emergency departments, supporting the value of continuous audit and education in strengthening airway management practices [7].

However, most published airway quality improvement studies have focused primarily on procedural outcomes, such as intubation success rates and peri-intubation complications, rather than evaluating healthcare professionals' knowledge of and adherence to contemporary evidence-based airway management guidelines. Moreover, evidence from low-resource settings, particularly regarding closed-loop clinical audits assessing adherence to the latest AHA ACLS airway management recommendations, remains limited [7].

Almanagil Teaching Hospital is a secondary care teaching hospital where physicians at different levels of training are actively involved in the emergency management of critically ill patients. Recognizing the absence of local data evaluating physicians' adherence to AHA airway management recommendations, a closed-loop clinical audit was selected as the quality improvement methodology because it enables systematic assessment of current practice against predefined standards, implementation of targeted interventions, and subsequent re-audit to determine whether measurable improvements have been achieved, thereby completing the audit cycle. Following completion of the initial audit cycle, targeted educational interventions were implemented to address the identified deficiencies. A second audit cycle was subsequently conducted to evaluate the effectiveness of these interventions and determine whether they resulted in sustained improvements in physicians' knowledge of and adherence to evidence-based airway management guidelines.

Therefore, the primary objective of this study was to evaluate the effectiveness of a multifaceted quality improvement intervention in improving physicians' knowledge of and adherence to the 2025 AHA ACLS airway management guidelines through a closed-loop clinical audit.

## Materials and methods

Study design and setting

A prospective closed-loop clinical audit was conducted at Almanagil Teaching Hospital, a secondary care teaching hospital in Sudan, to evaluate and improve healthcare professionals' knowledge of and adherence to evidence-based airway management principles during CPR. The audit was performed in accordance with the principles of clinical audit by comparing current practice against predefined standards, implementing targeted quality improvement interventions, and reassessing performance following their implementation.

The audit commenced on 1 March 2026. The first audit cycle was conducted over a two-week period from 1 March to 14 March 2026, during which baseline knowledge of and adherence to airway management principles were assessed. Following completion of the baseline assessment, a multifaceted quality improvement program was implemented over one month (15 March-14 April 2026). A prospective re-audit (second audit cycle) was subsequently conducted over two months from 15 April to 14 June 2026 using the same methodology and assessment criteria to evaluate the effectiveness and sustainability of the interventions.

Audit standards

The audit standards were derived from the 2025 AHA ACLS Guidelines and other internationally accepted recommendations for adult advanced life support [2]. Key standards included minimizing interruptions in chest compressions during airway interventions, correct application of basic airway maneuvers, effective bag-valve-mask ventilation, appropriate ventilation rates following advanced airway placement, use of continuous waveform capnography for airway confirmation, appropriate timing of advanced airway insertion, and comprehensive documentation of airway management procedures. Target compliance levels were established before data collection and ranged from 90% to 100%, depending on the specific audit criterion.

Study population and sampling

The study included house officers, medical officers, and registrars working at Almanagil Teaching Hospital who were directly involved in the emergency assessment and resuscitation of adult patients. Healthcare professionals from different clinical departments were included in the audit to provide a representative assessment of airway management knowledge across varying levels of clinical experience. Healthcare professionals who were not directly involved in the emergency assessment and resuscitation of adult patients, those who were absent during the data collection period (e.g., because of leave or clinical rotation), and those who declined to participate were excluded from the audit.

Sample size

A total coverage sampling approach was adopted for this clinical audit. All eligible house officers, medical officers, and registrars directly involved in the emergency assessment and resuscitation of adult patients during each audit cycle were invited to participate. Consequently, 50 healthcare professionals were included in the first audit cycle and 50 in the second audit cycle. The audit employed independent (unpaired) cross-sectional samples for the two audit cycles. Questionnaires were completed anonymously, and participants were not individually matched between the baseline and re-audit assessments. Participation was voluntary.

Data collection

Data were collected using a structured, self-administered questionnaire specifically designed to evaluate knowledge of and adherence to evidence-based airway management principles recommended by the AHA ACLS guidelines [2]. The questionnaire consisted of multiple-choice questions covering key domains of airway management, including basic airway assessment and airway-opening maneuvers, bag-valve-mask ventilation, advanced airway management, ventilation strategies during CPR, continuous waveform capnography, oxygen administration, recognition of poor airway management practices, airway adjuncts, and leadership and team communication during resuscitation (Appendix).

The same questionnaire was used during both audit cycles to ensure direct comparability of results. Prior to administration, the questionnaire was reviewed by senior clinicians with expertise in emergency medicine and resuscitation to ensure content validity and consistency with current ACLS recommendations [2].

Quality improvement intervention

Following completion of the baseline audit, a comprehensive quality improvement program was implemented to address the identified deficiencies. The intervention included structured educational lectures on adult airway management, interactive case-based discussions, practical demonstrations of basic and advanced airway techniques, dissemination of educational materials summarizing current ACLS recommendations, and regular feedback sessions highlighting the deficiencies identified during the first audit cycle.

Visual educational posters illustrating evidence-based airway management algorithms were displayed in emergency and critical care areas to reinforce learning during routine clinical practice. Healthcare professionals were encouraged to review current ACLS recommendations and participate actively in departmental educational activities throughout the intervention period.

Outcome measures

The primary outcome was the improvement in the proportion of participants correctly answering questionnaire items assessing airway management knowledge and adherence to ACLS recommendations between the first and second audit cycles.

Secondary outcomes included improvements in individual airway management domains, including recognition of appropriate airway maneuvers, ventilation strategies, capnography interpretation, advanced airway management, and understanding of CPR-related airway management principles.

Statistical analysis

Data were entered into Microsoft Excel and analyzed using IBM SPSS Statistics for Windows, Version 24.0 (IBM Corp., Armonk, NY, USA). Categorical variables were summarized as frequencies and percentages. The proportions of correct responses between the first and second audit cycles were compared using the chi-square test or Fisher's exact test, as appropriate. Statistical significance was defined as a two-sided p-value <0.05.

Ethical considerations

This clinical audit was conducted as part of the quality improvement program at Almanagil Teaching Hospital after obtaining institutional approval from the Clinical Audit Department and the hospital's Institutional Review Board (IRB Reference No. 2026-AI-033). The audit was performed in accordance with internationally recognized clinical governance frameworks and institutional ethical standards. Participation was voluntary, and written informed consent was obtained from all healthcare professionals before completion of the questionnaire. Data were collected anonymously, and participant confidentiality was strictly maintained throughout the study. As this project was conducted as a quality improvement clinical audit with no patient involvement or alteration to routine clinical care, no additional interventions were performed beyond the educational program. The study adhered to the ethical principles of the Declaration of Helsinki and complied with local institutional requirements for clinical audit and quality improvement.

## Results

A total of 100 healthcare professionals participated in the audit, with 50 participants included in each audit cycle. Comparison of the baseline and re-audit findings demonstrated marked improvements in knowledge of and adherence to evidence-based airway management principles following implementation of the quality improvement interventions (Table 1).

**Table 1:** Comparison of healthcare professionals' knowledge of and adherence to airway management principles before and after implementation of the quality improvement intervention Data are presented as n (%). Percentages were calculated using a denominator of 50 participants in each audit cycle. Comparisons between the first and second audit cycles were performed using the Pearson chi-square test. A two-sided p-value <0.05 was considered statistically significant. BVM, bag-valve-mask; CPR, cardiopulmonary resuscitation; ETCO₂, end-tidal carbon dioxide; OPA, oropharyngeal airway; NPA, nasopharyngeal airway

Airway management principle	First cycle, n (%)	Second cycle, n (%)	Improvement (%)	χ²	P-value
Minimizing interruption of chest compressions during intubation	33 (66.0)	48 (96.0)	+30.0	14.62	<0.001
Proper bag-valve-mask ventilation and airway positioning	43 (86.0)	48 (96.0)	+10.0	3.05	0.081
Correct ventilation rate with advanced airway	43 (86.0)	50 (100.0)	+14.0	7.53	0.006
Use of capnography for airway confirmation	33 (66.0)	49 (98.0)	+32.0	17.34	<0.001
Prioritization of high-quality chest compressions	35 (70.0)	48 (96.0)	+26.0	11.98	<0.001
Knowledge of the most critical determinant of survival during cardiac arrest	17 (34.0)	42 (84.0)	+50.0	25.84	<0.001
Knowledge of maximum recommended interruption time during CPR	23 (46.0)	49 (98.0)	+52.0	33.53	<0.001
Continuous chest compressions with advanced airway	4 (8.0)	34 (68.0)	+60.0	38.20	<0.001
Understanding of waveform capnography during CPR	27 (54.0)	41 (82.0)	+28.0	9.01	0.003
Appropriate timing of advanced airway placement	31 (62.0)	43 (86.0)	+24.0	7.14	0.008
Recognition of poor airway management practices	39 (78.0)	40 (80.0)	+2.0	0.06	0.806
Knowledge of harmful effects of hyperventilation	33 (66.0)	39 (78.0)	+12.0	1.79	0.181
Preferred airway maneuver in non-trauma patients	43 (86.0)	48 (96.0)	+10.0	3.05	0.081
Preferred airway maneuver in trauma patients	23 (46.0)	45 (90.0)	+44.0	22.24	<0.001
Knowledge regarding airway adjuncts (OPA/NPA)	21 (42.0)	41 (82.0)	+40.0	16.98	<0.001
Recommended oxygen concentration during BVM ventilation	50 (100.0)	50 (100.0)	0.0	-	-
Understanding of ETCO₂ significance during CPR	24 (48.0)	44 (88.0)	+40.0	18.38	<0.001
Importance of leadership and role assignment during resuscitation	39 (78.0)	49 (98.0)	+20.0	9.47	0.002

Statistically significant improvements were observed in most assessed domains. Knowledge of continuous chest compressions following advanced airway placement showed the greatest absolute improvement, increasing from four (8.0%) during the first cycle to 34 (68.0%) in the second cycle (χ²=38.20, p<0.001). Similarly, knowledge of the maximum recommended interruption time during CRP improved from 23 (46.0%) to 49 (98.0%) (χ²=33.53, p<0.001), while recognition of the most critical determinant of survival during cardiac arrest increased from 17 (34.0%) to 42 (84.0%) (χ²=25.84, p<0.001).

Significant improvements were also demonstrated in the use of capnography for airway confirmation (33 (66.0%) vs. 49 (98.0%); χ²=17.34, p<0.001), knowledge regarding airway adjuncts (21 (42.0%) vs. 41 (82.0%); χ²=16.98, p<0.001), understanding of the significance of end-tidal carbon dioxide (ETCO₂) during CPR (24 (48.0%) vs. 44 (88.0%); χ²=18.38, p<0.001), the preferred airway maneuver in trauma patients (23 (46.0%) vs. 45 (90.0%); χ²=22.24, p<0.001), minimizing interruptions in chest compressions during intubation (33 (66.0%) vs. 48 (96.0%); χ²=14.62, p<0.001), prioritization of high-quality chest compressions (35 (70.0%) vs. 48 (96.0%); χ²=11.98, p<0.001), understanding of waveform capnography during CPR (27 (54.0%) vs. 41 (82.0%); χ²=9.01, p=0.003), appropriate timing of advanced airway placement (31 (62.0%) vs. 43 (86.0%); χ²=7.14, p=0.008), correct ventilation rate following advanced airway placement (43 (86.0%) vs. 50 (100.0%); χ²=7.53, p=0.006), and the importance of leadership and role assignment during resuscitation (39 (78.0%) vs. 49 (98.0%); χ²=9.47, p=0.002).

In contrast, improvements in proper bag-valve-mask ventilation and airway positioning (43 (86.0%) vs. 48 (96.0%); χ²=3.05, p=0.081), the preferred airway maneuver in non-trauma patients (43 (86.0%) vs. 48 (96.0%); χ²=3.05, p=0.081), and knowledge of the harmful effects of hyperventilation (33 (66.0%) vs. 39 (78.0%); χ²=1.79, p=0.181) showed numerical improvement but did not reach statistical significance. Recognition of poor airway management practices remained largely unchanged between the two audit cycles (39 (78.0%) vs. 40 (80.0%); χ²=0.06, p=0.806). Knowledge of the recommended oxygen concentration during bag-valve-mask ventilation remained uniformly high, with all participants answering correctly in both audit cycles (50 (100.0%) in each cycle).

## Discussion

The present closed-loop clinical audit demonstrated that a structured, multifaceted educational intervention was associated with substantial improvements in healthcare professionals' knowledge of and adherence to evidence-based airway management principles. Significant improvements were observed in the majority of the assessed domains, particularly those related to minimizing interruptions in chest compressions, use of waveform capnography, continuous chest compressions following advanced airway placement, airway adjuncts, and recognition of key resuscitation principles. These findings suggest that targeted educational interventions combined with regular feedback can effectively improve clinicians' knowledge of airway management and promote greater adherence to contemporary resuscitation guidelines. Similar findings have been reported in recent international recommendations, which emphasize that continuous education, multidisciplinary team training, and competency-based airway programs are fundamental to maintaining high-quality airway management and improving patient safety [8].

The significant improvement observed across most airway management domains is likely attributable to the multifaceted educational intervention implemented between the two audit cycles. The intervention combined structured teaching sessions, case-based discussions, practical demonstrations, educational posters, and regular feedback, all of which are recognized as effective strategies for improving knowledge retention and guideline adherence [9,10]. Similar findings have been reported in recent studies evaluating simulation-based airway education [9,10]. Paliwal et al. demonstrated that structured simulation training significantly improved participants' adherence to difficult airway management algorithms, technical performance, and team coordination, with sustained improvements observed six months after training [9]. Likewise, a recent systematic review by Köhler et al. concluded that simulation-based airway management training consistently enhances healthcare professionals' confidence, knowledge, and preparedness, although continued reinforcement is required to maintain long-term competence [10]. These findings support the concept that repeated education combined with practical reinforcement is considerably more effective than traditional didactic teaching alone and likely explains the substantial improvements observed following the quality improvement intervention in the present audit [9,10].

Another important finding of the present audit was the significant improvement in participants' knowledge of continuous chest compressions following advanced airway placement, minimization of interruptions during intubation, and the appropriate use of waveform capnography. These improvements are clinically important because maintaining a high chest compression fraction while securing the airway is a fundamental principle of high-quality CRP [8,11]. The 2025 AHA Guidelines and the 2025 European Resuscitation Council (ERC) Guidelines continue to recommend minimizing interruptions in chest compressions, confirming endotracheal tube placement with continuous waveform capnography, and using capnography to monitor the quality of CPR and detect return of spontaneous circulation [8,11]. Furthermore, a recent analysis of the Pragmatic Airway Resuscitation Trial demonstrated that dynamic increases in ETCO₂ during resuscitation were significantly associated with return of spontaneous circulation, highlighting the clinical value of capnography beyond confirmation of airway placement [12]. Collectively, these findings support the substantial improvements observed in the present audit and reinforce the importance of incorporating capnography interpretation and uninterrupted CPR into routine airway management training programs [8,11-13].

Although most airway management domains demonstrated significant improvement following the quality improvement intervention, some areas, including proper bag-valve-mask ventilation and airway positioning, knowledge of the harmful effects of hyperventilation, the preferred airway maneuver in non-trauma patients, and recognition of poor airway management practices, did not reach statistical significance despite showing numerical improvements. One possible explanation is the relatively high baseline performance observed in these domains, leaving limited scope for further measurable improvement (a ceiling effect). In addition, competencies such as effective bag-valve-mask ventilation and recognition of airway errors require repeated hands-on practice and simulation-based reinforcement rather than knowledge-based teaching alone [14]. Recent evidence suggests that while educational interventions substantially improve theoretical knowledge and confidence, psychomotor airway skills and non-technical competencies, including situational awareness, communication, and decision-making under pressure, are better maintained through regular multidisciplinary simulation and repeated deliberate practice [14]. Similarly, the 2025 Difficult Airway Society guidelines emphasize that airway safety depends not only on technical expertise but also on human factors, structured team communication, cognitive workload management, and continuous competency assessment [8]. These observations may explain why certain domains improved less markedly than others and highlight the need for ongoing simulation-based education and periodic competency reassessment to achieve sustained improvements in airway management performance [8,14].

The findings of the present audit have important implications for clinical practice and quality improvement. Airway management is a time-critical intervention in emergency medicine, and deficiencies in clinicians' knowledge or technical performance may contribute to preventable adverse events during resuscitation [8]. The substantial improvements observed following the educational intervention suggest that regular audit combined with structured education can effectively strengthen adherence to evidence-based airway management principles. Importantly, the audit also demonstrates the value of the closed-loop clinical audit process as a sustainable quality improvement strategy, enabling healthcare institutions to identify deficiencies, implement targeted interventions, and objectively evaluate their effectiveness. Contemporary airway education increasingly advocates competency-based training supported by simulation, regular assessment, multidisciplinary teamwork, and continuous audit rather than reliance on one-time educational activities [10]. Incorporating these approaches into routine clinical practice may enhance clinicians' preparedness, improve compliance with international airway management guidelines, and ultimately contribute to safer patient care and improved resuscitation outcomes [8,10].

The findings of this audit are particularly relevant to healthcare systems in low- and middle-income countries, where limitations in equipment, simulation facilities, and access to structured airway training may contribute to variability in airway management practices [15]. In such settings, cost-effective quality improvement initiatives, including regular clinical audit, targeted educational programs, and multidisciplinary simulation, provide practical strategies for enhancing clinicians' competence without requiring substantial financial investment [15,16]. The sustained improvements observed following the present intervention support the role of closed-loop clinical audit as an effective and feasible quality improvement methodology for strengthening adherence to international airway management recommendations. Recent reviews have similarly emphasized that, in resource-limited settings, optimizing clinician education, standardizing airway protocols, promoting team-based training, and implementing regular audit and feedback are among the most effective interventions for improving airway safety and reducing preventable complications [14-16]. Therefore, integrating these strategies into routine institutional practice may facilitate long-term improvements in both clinician performance and patient safety during emergency airway management [15-17].

Strengths and limitations

This study has several strengths. To our knowledge, it is among the few closed-loop clinical audits evaluating healthcare professionals' knowledge of and adherence to evidence-based airway management principles in a Sudanese teaching hospital. The closed-loop audit design allowed objective assessment of baseline performance, implementation of targeted quality improvement interventions, and subsequent evaluation of their effectiveness using identical assessment criteria. Furthermore, the intervention incorporated multiple educational strategies, including structured teaching sessions, practical demonstrations, educational materials, and regular feedback, reflecting a pragmatic approach that can be readily implemented in similar healthcare settings with limited resources. These characteristics enhance the applicability and reproducibility of the intervention in routine clinical practice.

Nevertheless, several limitations should be acknowledged. First, this was a single-center study involving a relatively small sample of healthcare professionals, which may limit the generalizability of the findings to other institutions. Second, although the questionnaire underwent expert review to establish content validity and consistency with the 2025 AHA ACLS guidelines, formal psychometric validation and reliability testing were not performed. Third, the audit primarily evaluated participants' knowledge of and adherence to evidence-based airway management principles using a structured questionnaire rather than directly assessing clinical performance during real airway management scenarios. Therefore, improvements in questionnaire scores may not necessarily translate into equivalent improvements in procedural competence or patient outcomes. Fourth, the relatively short follow-up period did not permit evaluation of long-term knowledge retention or the sustainability of the observed improvements. Finally, although the educational intervention was associated with significant improvements across most assessed domains, the multifaceted nature of the intervention precluded determination of the individual contribution of each educational component. Future multicenter studies incorporating simulation-based competency assessment, direct observation of clinical performance, and longer follow-up periods are warranted to evaluate the durability of educational interventions and their impact on patient-centered outcomes.

## Conclusions

This closed-loop clinical audit demonstrated that a structured, multifaceted educational intervention was associated with significant improvements in healthcare professionals' knowledge of and adherence to evidence-based airway management principles as assessed by a structured questionnaire at Almanagil Teaching Hospital. Marked improvements were observed across most assessed domains, particularly those related to uninterrupted chest compressions, advanced airway management, waveform capnography, and key resuscitation principles. These findings highlight the effectiveness of combining targeted education, practical reinforcement, and regular feedback within a structured quality improvement framework to improve questionnaire-based knowledge of and adherence to guideline-based airway management principles. The audit also demonstrates the value of the clinical audit cycle as a practical and sustainable approach for identifying deficiencies, implementing evidence-based interventions, and evaluating their educational impact. Continued educational initiatives, periodic competency assessment, and regular re-audit are recommended to sustain these improvements. Future studies incorporating direct observation of clinical practice and patient-centered outcomes are warranted to determine whether these educational gains translate into sustained improvements in airway management performance and patient care.

## Appendices

Airway management knowledge questionnaire

Instructions

Please select the single best answer for each question. The questionnaire is based on the 2025 American Heart Association (AHA) Advanced Cardiovascular Life Support (ACLS) Guidelines and is intended to assess knowledge of evidence-based airway management during adult cardiopulmonary resuscitation [2].

1. During endotracheal intubation, what is the maximum recommended interruption in chest compressions?

A. 5 seconds

B. 10 seconds

C. 20 seconds

D. Until successful intubation

2. Which of the following represents proper bag-valve-mask (BVM) ventilation and airway positioning?

A. Hyperextend the neck in all patients

B. Maintain an open airway using appropriate positioning and deliver effective BVM ventilation

C. Ventilate as rapidly as possible

D. Delay ventilation until intubation

3. After placement of an advanced airway during CPR, what is the recommended ventilation rate?

A. 6 breaths/min

B. 10 breaths/min

C. 20 breaths/min

D. 30 breaths/min

4. What is the recommended method for confirming endotracheal tube placement?

A. Chest auscultation only

B. Chest X-ray

C. Continuous waveform capnography

D. Observation of chest movement alone

5. During cardiac arrest, which intervention should always be prioritized?

A. Early endotracheal intubation

B. Defibrillation only

C. High-quality chest compressions

D. Administration of oxygen

6. What is the single most important determinant of survival during cardiac arrest?

A. Advanced airway placement

B. High-quality CPR with early defibrillation when indicated

C. Intravenous access

D. Medication administration

7. What is the maximum recommended interruption in chest compressions during airway placement?

A. Less than 10 seconds

B. Less than 20 seconds

C. Less than 30 seconds

D. No limit

8. Once an advanced airway has been inserted, chest compressions should be:

A. Interrupted during each ventilation

B. Continuous while asynchronous ventilations are delivered

C. Stopped every two minutes

D. Performed only after ventilation

9. What is the primary role of waveform capnography during CPR?

A. Measure oxygen saturation

B. Confirm airway placement and monitor CPR quality

C. Assess blood pressure

D. Detect arrhythmias

10. When should an advanced airway be considered during adult CPR?

A. Immediately for every patient

B. Only after return of spontaneous circulation

C. When indicated without interrupting high-quality CPR

D. Only after all medications have been given

11. Which of the following is considered poor airway management practice?

A. Confirming tube placement with capnography

B. Minimizing interruptions in chest compressions

C. Excessive interruption of CPR during airway management

D. Continuous monitoring of ventilation

12. Which complication may result from hyperventilation during CPR?

A. Increased coronary perfusion

B. Reduced venous return and decreased cardiac output

C. Improved cerebral perfusion

D. Faster return of spontaneous circulation

13. In an unconscious patient without suspected cervical spine injury, the preferred airway-opening manoeuvre is:

A. Jaw-thrust

B. Head tilt-chin lift

C. Triple airway manoeuvre

D. None of the above

14. In a trauma patient with suspected cervical spine injury, the preferred airway-opening manoeuvre is:

A. Head tilt-chin lift

B. Jaw-thrust

C. Neck extension

D. Chin lift only

15. Which airway adjunct may be used to maintain airway patency in an unconscious patient without a gag reflex?

A. Oropharyngeal airway (OPA)

B. Nasopharyngeal airway (NPA)

C. Both as clinically appropriate

D. Neither

16. During bag-valve-mask ventilation in adult cardiac arrest, what oxygen concentration should be used when available?

A. 21%

B. 40%

C. 60%

D. 100%

17. During CPR, what does a sudden increase in end-tidal carbon dioxide (ETCO₂) most likely indicate?

A. Hyperventilation

B. Airway obstruction

C. Return of spontaneous circulation (ROSC)

D. Equipment malfunction

18. What is the primary role of the team leader during resuscitation?

A. Perform all procedures personally

B. Coordinate the team, assign roles, and ensure adherence to resuscitation protocols

C. Administer medications only

D. Document the resuscitation
